# Both telomeric and non-telomeric DNA damage are determinants of mammalian cellular senescence

**DOI:** 10.1186/1756-8935-1-6

**Published:** 2008-11-03

**Authors:** Asako J Nakamura, Y Jeffrey Chiang, Karen S Hathcock, Izumi Horikawa, Olga A Sedelnikova, Richard J Hodes, William M Bonner

**Affiliations:** 1 Laboratory of Molecular Pharmacology, National Cancer Institute, National Institutes of Health, Rockville Pike, Bethesda, MD 20892, USA; 2 Experimental Immunology Branch, National Cancer Institute, National Institutes of Health, Rockville Pike, Bethesda, MD 20892, USA; 3 Laboratory of Biosystems and Cancer, National Cancer Institute, National Institutes of Health, Rockville Pike, Bethesda, MD 20892, USA; 4 National Institute on Aging, National Institutes of Health, Rockville Pike, Bethesda MD 20892, USA

## Abstract

**Background:**

Cellular senescence is a state reached by normal mammalian cells after a finite number of cell divisions and is characterized by morphological and physiological changes including terminal cell-cycle arrest. The limits on cell division imposed by senescence may play an important role in both organismal aging and in preventing tumorigenesis. Cellular senescence and organismal aging are both accompanied by increased DNA damage, seen as the formation of γ-H2AX foci (γ-foci), which may be found on uncapped telomeres or at non-telomeric sites of DNA damage. However, the relative importance of telomere- and non-telomere-associated DNA damage to inducing senescence has never been demonstrated. Here we present a new approach to determine accurately the chromosomal location of γ-foci and quantify the number of telomeric versus non-telomeric γ-foci associated with senescence in both human and mouse cells. This approach enables researchers to obtain accurate values and to avoid various possible misestimates inherent in earlier methods.

**Results:**

Using combined immunofluorescence and telomere fluorescence *in situ *hybridization on metaphase chromosomes, we show that human cellular senescence is not solely determined by telomeric DNA damage. In addition, mouse cellular senescence is not solely determined by non-telomeric DNA damage. By comparing cells from different generations of telomerase-null mice with human cells, we show that cells from late generation telomerase-null mice, which have substantially short telomeres, contain mostly telomeric γ-foci. Most notably, we report that, as human and mouse cells approach senescence, all cells exhibit similar numbers of total γ-foci per cell, irrespective of chromosomal locations.

**Conclusion:**

Our results suggest that the chromosome location of senescence-related γ-foci is determined by the telomere length rather than species differences *per se*. In addition, our data indicate that both telomeric and non-telomeric DNA damage responses play equivalent roles in signaling the initiation of cellular senescence and organismal aging. These data have important implications in the study of mechanisms to induce or delay cellular senescence in different species.

## Background

Normal mammalian cells have a finite replicative lifespan. After a certain number of cell divisions *in vitro*, these cells undergo a process known as cellular senescence, which is characterized by an irreversible cell-cycle arrest accompanied by other physiological and morphological changes [1,2]. Cellular senescence is important for preventing tumorigenesis *in vivo *and in addition may play a role in organismal aging [3,4]. There is considerable evidence suggesting that accumulation of DNA damage plays a critical role in both *in vitro *senescence and *in vivo *aging [5-9].

One category of senescence-associated DNA damage that has received a great deal of attention is the damage response associated with telomere shortening and consequent telomere dysfunction or uncapping [10]. It has been shown that DNA repair proteins, including γ-H2AX [11,12], are localized at uncapped telomeres [13]. This telomeric DNA damage response has also been shown to be a potential inducer of senescence or cell death [5-7], as well as of *in vivo *aging in both model systems and human pathology [3]. Therefore, it has been proposed that replicative cellular senescence is induced by telomere dysfunction [5-7,14].

However, there is considerable evidence that cellular senescence and organismal aging can occur through mechanisms other than telomere dysfunction [15-17]. For example, cells of laboratory mice, which have long telomeres, reach senescence in culture without apparent telomere uncapping [18]. The time necessary to reach senescence is increased when the cultures are maintained in a reduced (3%) oxygen atmosphere, suggesting that oxidative stress is involved [19].

Total numbers of DNA damage foci were found to increase similarly in both human and mouse cells during *in vivo *aging and during *in vitro *culture-induced cellular senescence [8,9]. Given the previous observation that telomeric foci are substantially more frequent in human than in mouse cells, these findings suggest that the overall DNA damage foci observed with aging and senescence may also include those with telomere-independent origins. Therefore, a complete understanding of the factors affecting senescence and aging requires knowledge of the relative contributions of telomeric and non-telomeric DNA damage.

In order to understand the relationship between these two types of DNA damage and *in vivo *and *in vitro *aging, we applied a technique that directly reveals the position of γ-foci on chromatids in metaphase spreads of human and mouse cells and simultaneously assesses the condition of the telomeres through telomere-fluorescence *in situ *hybridization (FISH) [20]. This technique permits localization of γ-foci to either the chromatid arms, corresponding to non-telomeric DNA damage, or to the end of the chromatid, corresponding to telomere damage. Telomere-FISH carried out in parallel provides a useful indication of the status of telomere shortening of these chromatids. Using this technique, we confirm our previous findings that DNA damage accumulates similarly in both human and mouse cells during the replicative lifespan [8] and that the total number of γ-foci appeared to be a consistent characteristic of the senescent state. In addition, by comparing cells from different generations of telomerase-null mice, we show that telomere length, rather than other differences between human and mouse, determines the differential pattern of senescence-associated DNA damage. This study demonstrates that the mechanism(s) of senescence-associated DNA damage involve both telomere- and non-telomere-associated foci, with the distribution of these two components dependent, to a large degree, upon the length and presumably, the functionality of the telomeres.

## Results

### Human and mouse primary cells show different patterns of senescence-related γ-foci

**Figure 1:**
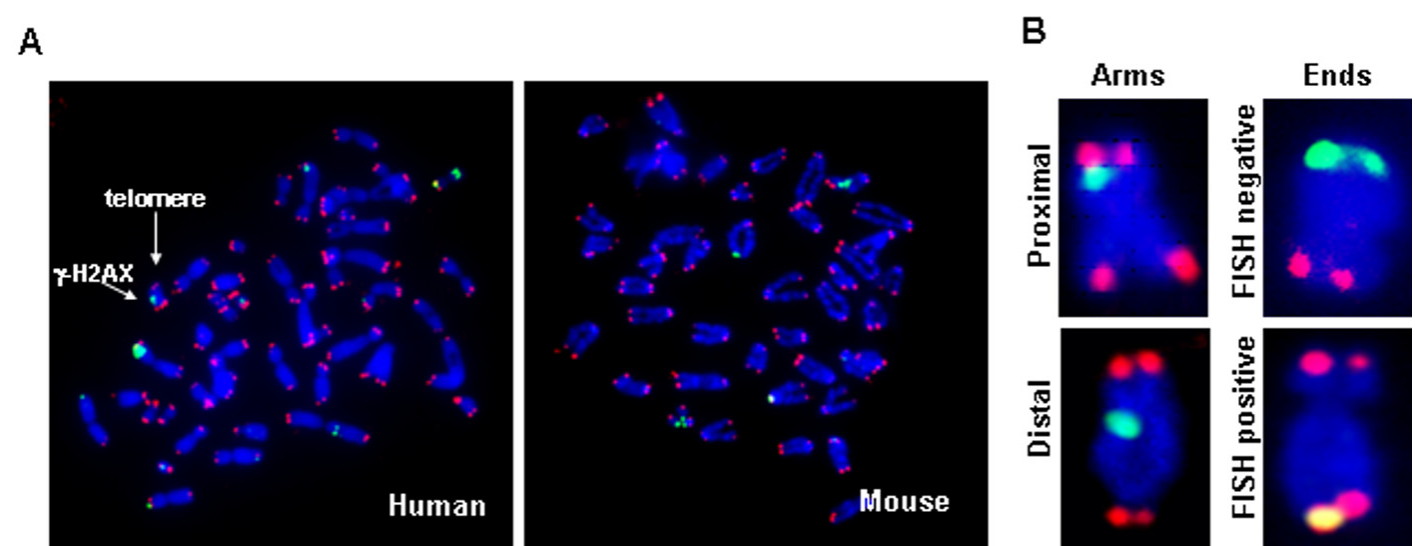
**γ-H2AX immunostaining on metaphase chromosomes**. (A) Typical metaphase spreads of human (left) and mouse (right) fibroblasts stained for γ-H2AX (green) and telomeric DNA (red). (B) Scoring of foci as along the chromatid arms, proximal or distal to the telomeres, or on the chromatid ends, fluorescence *in situ *hybridization negative or positive.

Metaphase chromosome spreads were prepared from cultures of human and mouse cells at low population doubling (PD) times and during the penultimate PD before the onset of senescence (hereafter termed pre-senescent) (experimental details are described in Additional file 1). Senescence status was defined by both growth arrest and senescence-associated β-galactosidase (SA-β-gal) staining assay (> 90% SA-β-gal positive cells in human cultures, and > 80% SA-β-gal positive cells in mouse cultures) (Additional files 2A and 2B). Metaphase chromosomes were stained for γ-H2AX and for telomeric DNA in order to measure the numbers of uncapped telomeres and other types of DNA double-strand breaks (DSBs) (Figure 1A and 1B) [20]. Telomere-FISH/γ-H2AX staining of metaphase spreads permits visualization of the precise location of γ-foci on the chromosomes and their classification (Figure 1B), including those at the chromosomal ends lacking detectable telomere repeats (Figure 1B, FISH negative). The colcemid treatment did not affect γ-foci formation (Additional file 2C).

**Figure 2:**
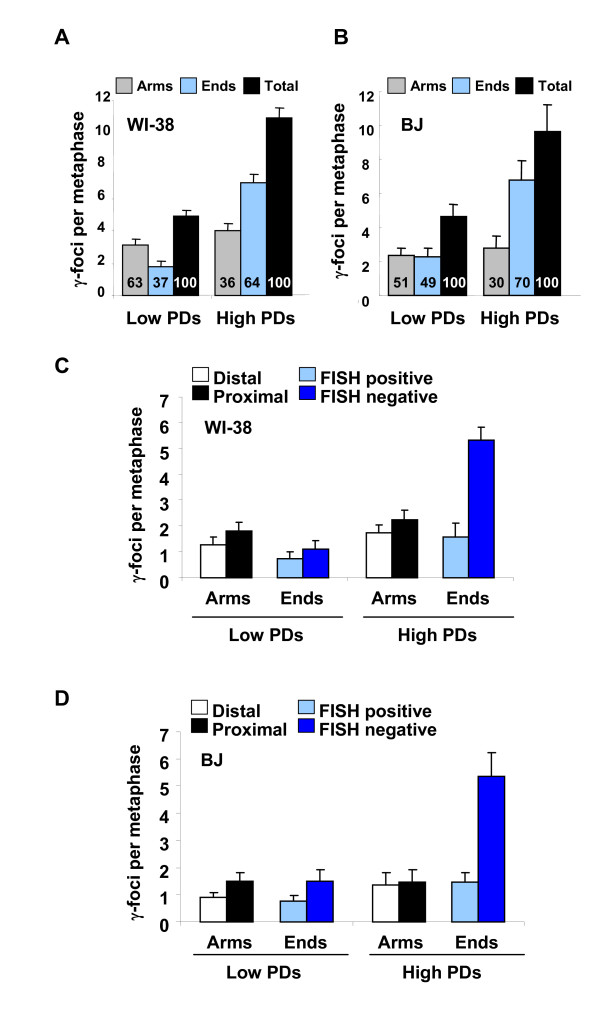
**Distribution of senescence-related γ-foci in human fibroblasts**. (A-B) Distribution of γ-foci on the metaphases of low and high population doublings (PDs) (pre-senescent), human embryo lung fibroblast (WI-38) (A) and foreskin fibroblast (BJ) (B). Proportion (%) of each type of damage is shown in each graph bar. Scoring is as in Figure 1B. (C-D) Scoring of the γ-foci as along the chromatid arms proximal to the telomere, along the chromatid arms distal to the telomeres, on the chromatid ends with fluorescence *in situ *hybridization (FISH) signal or on the chromatid ends without FISH signal. Low and high PDs of WI-38 cells (C) and BJ cells (D). On average more than 10 metaphases were screened per point in independent experiments. Error bars signify standard errors.

Cultures of two proliferating normal human fibroblast strains, lung WI-38 and skin BJ, exhibited increasing numbers of total γ-foci over time (Figure 2A and 2B, black bars). The technique revealed that at low PDs, the numbers of non-telomeric foci were greater than or equal to the numbers of telomeric foci, however, after increasing PDs and cumulative cell divisions, the foci were primarily telomeric, accounting for about 70% of the total number of foci in pre-senescent cultures (Figure 2A and 2B, blue bars). Among the total γ-foci at the chromatid ends in pre-senescent WI-38 and BJ cells, most of the γ-foci were negative for FISH signal (Figure 2C and 2D, dark blue bars), suggesting that these γ-foci were at critically short, dysfunctional telomeres. Interestingly, pre-senescent WI-38 cells contain fewer telomeric foci and more non-telomeric foci than do BJ cells. These results suggest that the WI-38 lung fibroblasts may be more susceptible to non-telomeric DNA damage in agreement with previous studies based on stress-related protein induction [21]. These results indicate that reliance on telomere-FISH or telomere-associated proteins in interphase cells to determine γ-focal positioning potentially underestimates the numbers of telomeric γ-foci [8,14,22,23]. These results confirm that during senescence in human cells, telomeric DNA damage increases while the non-telomeric DNA damage is already present in low PD cultures and appears to be a characteristic of the specific cell line. Our recently published analysis comparing γ-focal distribution in metaphases of young and senescent primary fibroblasts from a normal and a Werner syndrome (WS) donor showed a similar pattern of increased telomere-associated γ-foci during cellular senescence and in WS cultures [9]. This confirms that increasing telomere dysfunction plays a primary role in producing senescence- and age-associated DNA damage in humans.

**Figure 3:**
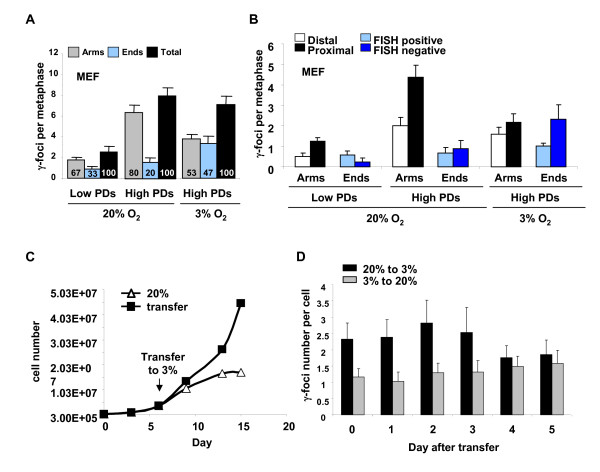
**Distribution of senescence-related γ-foci in mouse embryo fibroblasts**. (A) Distribution of γ-foci on metaphases of primary mouse embryonic fibroblasts (MEF), low population doublings (PDs), high PDs in 20% O_2 _and 3% O_2 _(see Additional file 1 for experimental details). Proportion (%) of each type of damage is shown in each graph bar. Scoring is as in Figure 1B. (B) Scoring of γ-foci as along the chromatid arms proximal to telomere, along the chromatid arms distal to the telomeres, on the chromatid ends with fluorescence *in situ *hybridization (FISH) signal or on the chromatid ends without FISH signal. On average more than 10 metaphases were screened per point in independent experiments. (C) MEFs cultured in 20% O_2 _(triangle) were shifted at day 5 to 3% O_2 _(square), or maintained in 20% O_2_. The average of two cultures is shown. Note that the cultures transferred from 20% to 3% O_2 _in panel A were already pre-senescent, and also became senescent in 3% O_2_. The cultures transferred from 20% to 3% O_2 _in this panel were low PDs and accelerated growth in 3% O_2_. (D) Number of γ-foci in MEFs after oxygen transfer is shown. See Additional file 3 for experimental details. Error bars signify standard errors.

Mouse embryonic fibroblast (MEF) cultures also exhibited increasing total numbers of γ-foci as they proliferated (Figure 3A, 20% O_2_, black bars). In low PD MEFs, non-telomeric foci outnumbered telomeric foci similar to human cells. However, at high PDs, the increased foci were primarily non-telomeric, accounting for approximately 80% of the total focal numbers in pre-senescent cultures (Figure 3A, 20% O_2_, gray bars). It has been shown that normoxic conditions are stressful to MEF growth, leading to earlier senescence, which can be lessened by growth in 3% O_2 _[16,19]. When pre-senescent MEFs growing in normoxic conditions were transferred to a 3% O_2 _atmosphere at seven PDs and cultured to the penultimate PD (experimental details are described in Additional file 1), the number of γ-foci along the chromatid arms significantly decreased (Figure 3A, 3% O_2_, gray bar), supporting the notion that a sizeable fraction of non-telomeric foci were caused by oxidative stress and that this stress plays a primary role in cellular senescence in mouse cells. MEFs growing in 3% O_2 _exhibited increased numbers of γ-foci on chromosome ends (Figure 3A, 3% O_2_, blue bar). This result suggested that these telomeres are dysfunctional, a surprising result for mouse cells. However, this notion was confirmed by the result that over 66% of these telomeric γ-foci were FISH negative (Figure 3B, dark blue bars), independent evidence from the γ-foci that these telomeres may be dysfunctional. Growth in 3% O_2 _permits additional cell replication (experimental details are described in Additional file 1) with accompanying telomere shortening, suggesting that shortened telomeres might accumulate during this time. The effects of culturing at low oxygen on MEF proliferation and on the number of DNA damage were monitored daily after transfer (Figure 3C and 3D), (experimental details are described in Additional file 3). When MEFs at three PDs were transferred from 20% O_2 _to 3% O_2_, cell growth accelerated (Figure 3C) and the total number of γ-foci decreased. In contrast, MEFs transferred from 3% to 20% exhibited increased numbers of γ-foci (Figure 3D). These findings again suggest that a sizeable fraction of DNA damage was caused by oxidative stress.

**Figure 4:**
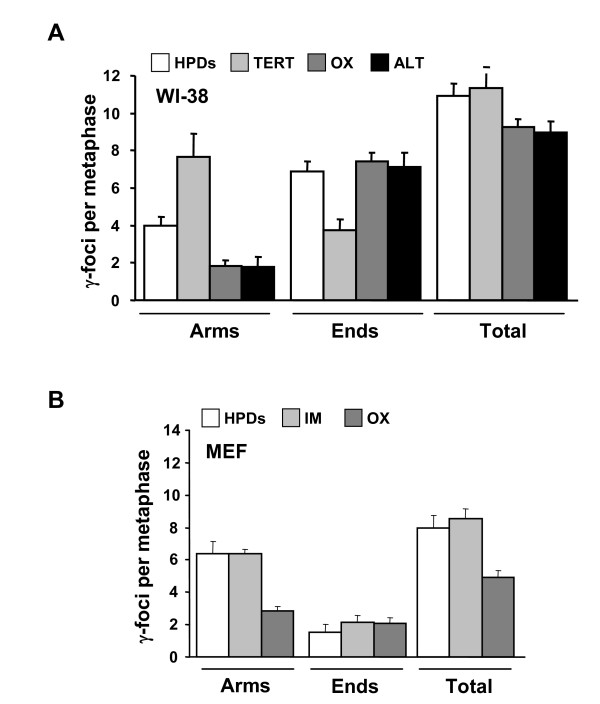
**Origins of senescence-associated γ-foci**. (A) Distribution of γ-foci in high population doubling (PD) WI-38 cells, after the expression of telomerase (TERT), after culturing with the 50 μM antioxidant tempol (OX), or in VA-13 with alternative lengthening of telomeres (ALT). (B) Distribution of γ-foci in high PD mouse embryonic fibroblast (MEF) cells, in spontaneously immortalized MEF cells (IM) or after culturing with the 50 μM tempol (OX). Scoring is as in Figure 1B. On average more than 10 metaphases were screened per point in independent experiments. Error bars signify standard errors.

To examine the nature of the γ-foci on the chromosome arms and chromatid ends more directly, we introduced telomere reverse transcriptase (TERT) in pre-senescent WI-38 cell populations. TERT expression led to an elongation of telomeres (data not shown) and to a decrease in the proportion of γ-foci on the chromatid ends in the human WI-38 cells, consistent with the idea that γ-foci localized to uncapped telomeres (Figure 4A, Ends, compare high PDs with TERT). In contrast to human cells, spontaneously immortalized mouse cells [24] displayed no change in the already small proportion of γ-foci at telomeres (Figure 4B, Ends, compare high PDs with immortalized MEF (IM)). These results support the hypothesis that most if not all γ-foci on the chromosome ends are due to telomere dysfunction.

Telomere length can be maintained in the absence of telomerase by a process termed alternative lengthening of telomeres (ALT) [25]. When VA-13 cells, a SV40-transformed variant of WI-38, which exhibits the ALT phenotype, were examined, the proportion of γ-foci on the chromatid ends was similar to that of pre-senescent WI-38 and higher than that of TERT-transduced WI-38 (Figure 4A, Ends, compare high PDs, TERT and ALT). Interestingly, about half of the γ-foci on the chromatid ends in VA-13 cells were FISH positive (see Additional file 4), suggesting that in these human cells, telomeres maintained by ALT are recognized as DNA damage even if sufficiently long to be scored as FISH positive.

In the second procedure, we added the antioxidant tempol (4-hydroxy-2,2,6,6-tetramethylpiperidine-N-oxyl), known to increase the lifespan of mice [26,27], to cell cultures nearing senescence (experimental details are described in Additional file 1). There was a substantial decrease in the total number of γ-foci in mouse cell cultures grown for 48 hours in medium including tempol (Figure 4B, Total, compare high PDs at normal conditions (high PDs) with antioxidant tempol (OX)). This decrease in the total number of γ-foci was caused by a significant decrease in the fraction of non-telomeric foci (Figure 4B, Arms, compare high PDs with OX). In addition, SA-β-gal positive cells decreased after the tempol treatment (see Additional file 2B). In human cell cultures, the presence of tempol also led to decreased numbers of non-telomeric γ-foci (Figure 4A, Arms, compare high PDs with OX) and to slight decreased number of SA-β-gal positive cells (see Additional file 2A). These results indicate that a sizeable fraction of γ-foci on the chromatid arms are of oxidative origin. In contrast, the presence of tempol did not affect the numbers of telomeric γ-foci in either human or mouse cell cultures (Figure 4A and 4B, Ends, compare high PDs with OX).

### Different telomere lengths in telomerase-null mouse cells lead to different patterns of γ-foci

**Figure 5:**
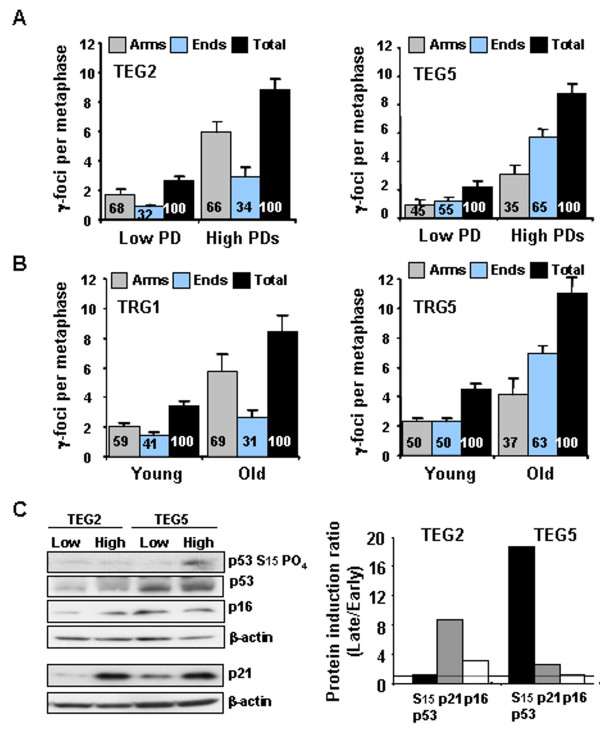
**Effect of telomerase deficiency on the distribution of senescence-related γ-foci and activation of damage signaling**. (**A) **Distribution of γ-foci in low population doubling (PD) and pre-senescent mouse embryonic fibroblasts (MEFs) from second (TEG2) and fifth (TEG5) generation telomere reverse transcriptase (TERT)-null mice. (B) Distribution of γ-foci in splenic lymphocytes taken from young and old first (TRG1) generation mTR-null mice, and from young and old fifth (TRG5) generation mTR-null mice. Scoring is as in Figure 1B. On average more than 10 metaphases were screened per point in independent experiments. Error bars signify standard errors. (C) Levels of DNA damage checkpoint proteins in early and high PDs MEFs from second (TEG2) and fifth (TEG5) generation TERT-null mice. Relative induction in senescent compared with low PD cells of the proteins shown in the left panel. Dashed line denotes no induction.

To determine whether the observed differences between patterns of γ-foci in human versus mouse cells might be due solely to differences in telomere length or whether other differences are involved, we utilized cells from mice lacking either the gene for mTERT or the gene for mouse telomerase RNA template (mTR). In these mice, each successive generation contains shorter telomeres until phenotypic effects of uncapped telomeres similar to those found in human cells appear in the fourth (G4) or fifth (G5) generation [28,29]. Comparison of γ-foci patterns between early generation mice and late generation mice can address the contribution of different length of telomeres to the distribution of γ-foci in the same species. MEFs were generated from second (G2) and fifth (G5) generation TERT-null mice grown to senescence. In metaphase spreads from pre-senescent G2 mouse cells, 66% of the γ-foci were on the chromatid arms and 34% were on the ends (Figure 5A, TEG2, high PDs), a senescence-related γ-focus distribution pattern similar to that observed in wild-type MEFs (Figure 3A, 20% O_2_, high PDs). In contrast, in metaphase spreads from pre-senescent G5 MEFs, 35% of the γ-foci were on the chromatid arms and 65% on the ends (Figure 5A, TEG5, high PDs), a pattern similar to that found in normal human cells (Figure 2A and 2B, high PDs).

An important biological question concerns the relationship between *in vitro *senescence and *in vivo *aging. We therefore analyzed young and old G1 and G5 mTR-null mice, in order to compare the effect of *in vivo *aging of splenic lymphocytes with that of *in vitro *MEF senescence discussed above. Splenic lymphocytes were obtained from these mice, stimulated to enter the cell cycle, and harvested at mitosis for metaphase spreads. Results obtained were similar to those obtained with MEFs that had undergone senescence *in vitro *(Figure 5B, compare with 5A). The increase in total γ-foci in old compared with young mTR-null mice (Figure 5B, black bars) was similar to that between early and high PDs MEFs from TERT-null mice (Figure 5A, black bars). In addition, the distribution of foci between the chromatid arms and ends was affected by aging in a manner similar to the observed effect of long-term culturing of MEFs (Figure 5A and 5B, gray and blue bars). In lymphocytes from old G1 mTR-null mice, 69% of the γ-foci were found on the chromatid arms (Figure 5B, TRG1, Old), similar to the 66% found in pre-senescent G2 TERT-null MEFs (Figure 5A, TEG2, high PDs) and pre-senescent wild-type MEFs (Figure 3A, 20% O_2_, high PDs). In contrast, in lymphocytes from old G5 mTR-null mice, only 37% of the γ-foci were found on the chromatid arms (Figure 5B, TRG5, Old), similar to the fractions found for the pre-senescent G5 TERT-null MEFs (Figure 5A, TEG5, high PDs) and the human cells (Figure 2A and 2B, high PDs), where the majority of γ-foci were at the chromatid ends. When telomere lengths were measured in wild-type and different generations of TERT-null mice, they were found to decrease with each generation and to be inversely proportional to the fraction of γ-foci on the chromatid ends (data not shown) [29]. These findings indicate that the different patterns of γ-focus distribution are due primarily to telomere length and not other mouse-human species differences. Importantly, these findings indicate that a similar distribution of γ-foci occurs in mouse cells during *in vitro *senescence as well as with *in vivo *aging.

The senescence-related, DNA-damage response involves two pathways, p21–p53 and p16-Rb. It has been suggested that the p21–p53 pathway is the main trigger for telomere-related senescence, while the p16-Rb pathway initiates stress-induced senescence [2,21,30]. To investigate differences in the activation of these damage signaling pathways in G2 and G5 TERT-null MEFs, we measured the induction of several key DNA damage and cell cycle checkpoint proteins (Figure 5C). Interestingly, p53, p21 and p16 levels were all spontaneously higher in G5 TERT-null MEF cells than in G2 cells (Figure 5C, compare TEG2 low with TEG5 low), suggesting that cumulative generations of breeding activate the checkpoint pathways in telomerase-deficient mice. However, serine-15 phosphorylation of p53 was extensive in TERT-null senescent MEFs from G5 mice but not in those from G2 mice (Figure 5C, p53 S^15 ^PO_4_). These results substantiate that the p53 pathway, which is important in telomere-related cellular senescence in human cells [7,21], is also activated in senescent MEFs from late generation TERT-null mice. In contrast, p16 was induced in senescent G2 but not G5 cells (Figure 5C, p16), suggesting that p16 may be less important in telomere-related senescence [31]. p21 levels increased in both the G5 and G2 senescent MEFs (Figure 5C, p21), suggesting that p21 could be induced by both telomeric and non-telomeric DNA damage signals in mouse cells [32]. These findings suggest that, in addition to differences in patterns of γ-focus distribution between early and late-generation telomerase-null mice, DNA damage pathways leading to cellular senescence also differ primarily as a consequence of different telomere lengths.

### Senescent mammalian cells contain a similar number of γ-foci irrespective of origin

**Figure 6:**
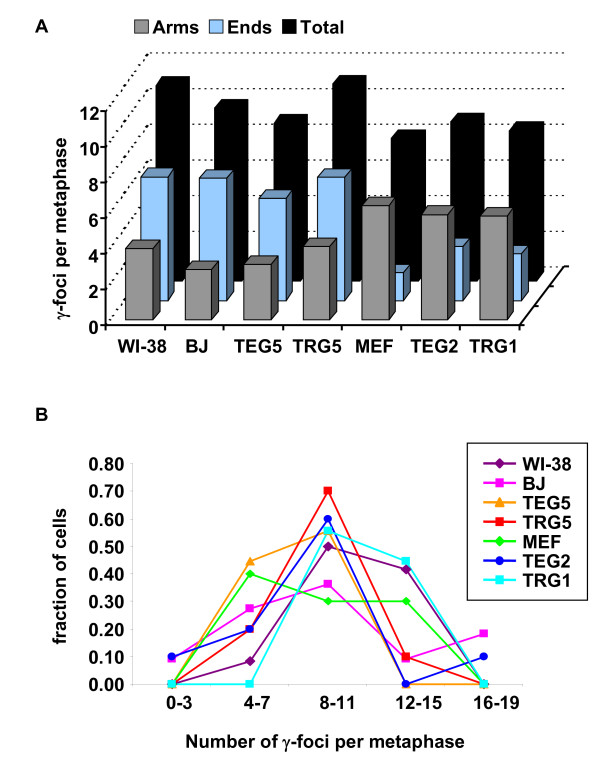
**Senescence in mammalian cells is associated with a set number of γ-foci regardless of origin**. (A) Numbers of γ-foci in the high population doublings or old cultures of the primary cell strains are arranged to show that the average total numbers of γ-foci per senescent or old cell are in the range 8–11, while the numbers of γ-foci found on the chromosome ends or on the arms are more variable. (B) The fractions of metaphase cells containing each range of γ-focal numbers are presented. Ranges of γ-focal numbers are taken to smooth the data.

In this study, we analyzed the localization of γ-foci in cell populations including those undergoing *in vitro *senescence and those undergoing *in vivo *aging. All the cell populations in the penultimate PDs *in vitro *or from *in vivo *aged mice, exhibited similar numbers of total γ-foci, even though the distribution of γ-foci on the chromatid ends and arms differed widely (Figure 6A). The total average numbers of γ-foci fall in a window of 8 to 11 γ-foci per metaphase, grouping around a mean of 9.5 (Figure 6B) and with a distribution similar to a Poisson distribution calculated on that mean, suggesting that the process may be partially stochastic. Thus we propose that senescence in mammalian cells *in vivo *and *in vitro *is associated with a set number of DNA lesions identified as γ-foci in a cell population and that the number, rather than the origin, of the lesions is the important determinant of senescence.

## Discussion

Since Hayflick and Moorhead [1] reported that normal human cells in culture are limited in replicative capacity to a finite number of PDs, efforts have been made to elucidate the mechanism behind this process and to relate it to organismal aging. That DNA damage plays an important role in aging is evidenced by the finding that premature aging syndromes, such as WS, ataxia telangiectasia, and Bloom syndrome, are defective in DNA DSB repair [3]. Recently we demonstrated that delayed accumulation of DNA DSB repair foci has been detected in primary cells from aged donors and WS donors as well as in senescent cells in culture [9]. These findings suggest that altered DNA DSB damage responses are a common characteristic in cellular and organismal aging. In fact, many studies have shown that DNA DSBs marked by γ-foci accumulate during *in vivo *aging and *in vitro *senescence [5-9,14]. These studies also suggested that senescence can be induced by the accumulation of DNA damage. Telomere shortening, which occurs during cell replication, is one of the factors that contribute to senescence- and age-associated DNA damage in humans. Telomeres that are critically short become functionally 'uncapped' and exhibit DNA damage γ-foci [13]. However, telomere shortening is not a major factor in the senescence of normal mouse cells whose telomeres are sufficiently long to avoid uncapping [18]. It has been shown that non-telomeric DNA damage initiated by environmental stresses such as atmospheric oxygen can be important senescence inducers in mouse cells [19]. Thus, it appears that an accumulation of different types of DNA damage lead to senescence in human and mouse cells. Although it has been suggested that different mechanisms of senescence may operate in human and mouse cells, the relative contributions of these two types of DNA damage have never been quantified. Previous studies have utilized techniques that fail to measure accurately the relative contributions of telomere-associated and non-telomere-associated DNA damage in cellular and organismal aging. A number of studies have relied upon double-staining of interphase cells for γ-H2AX and telomeric DNA sequences or the telomere-associated protein TRF2 [8,14,22,23]. However, while these studies demonstrated the presence of γ-foci on telomeres in interphase cells, the numbers were underestimated because γ-foci on telomeres too short to be visualized by staining for telomeric DNA or TRF2 were scored as non-telomeric. In fact, the present study revealed that most of the γ-foci at the chromatid ends in pre-senescent human cells were telomere-FISH negative (Figure 2C and 2D). Therefore, the efficiency of detecting telomeric repeats might be different depending on cell type (interphase versus metaphase) and previous studies might have underestimated the association of γ-foci with extremely short telomeres lacking telomeric-FISH signals or TRF2 foci. In addition, while chromatin immunoprecipitation/micro-array studies using chromosome panels unequivocally revealed γ-H2AX bound to telomeric and subtelomeric sequences [5], other non-telomeric genomic sequences may not bind sufficient γ-H2AX to allow detection, leading to underestimation of non-telomeric γ-H2AX.

In this study, we analyzed the location of sites of DNA DSB damage along the chromosomes of mammalian cells undergoing senescence in culture, using methodology capable of rigorously distinguishing telomeric from non-telomeric DNA damage. We demonstrated that senescence-related γ-foci in human cells are mostly telomeric, whereas foci in normal mouse cells are mostly non-telomeric. These results for the first time provide a quantitative measure of the amounts of telomere-related and telomere-independent DNA damage present in senescent cells. The ability to distinguish between these two types of γ-foci is also important in that it allows assessment of whether each type of focus can be prevented or resolved. As shown in Figures 3 and 4, the number of non-telomeric DNA DSBs induced by reactive oxygen species was decreased by culture in low oxygen, suggesting that this type of DNA damage may be reversible. As the rate of shortening of telomeric DNA can be slowed under conditions of low oxygen [33], the accumulation of telomeric damage may also be prevented or retarded, although resolution of telomeric damage, once formed, may require the action of telomerase.

The presence of γ-foci has been detected on mitotic chromosomes before and after anaphase, indicating checkpoint adaptation, which has been described previously in yeast cells, occurs in human cells [34-36]. Checkpoint adaptation was originally described in yeast as the ability to divide following a sustained checkpoint arrest in the presence of unrepairable DNA breaks. Human osteosarcoma cells also enter mitosis with γ-foci, suggesting that human cells are able to exit the checkpoint arrest and divide before the damage has been fully repaired. In addition, the G2/M checkpoint seems to have a defined threshold of 10 to 20 DSBs [37,38]. These findings strongly support the hypothesis that mammalian cells can enter into next cell cycle until DSBs accumulate to a threshold level and senescence-related DNA damage signals (p53-p21 pathway and/or Rb-p16 pathway) are fully activated to arrest the cell cycle.

It is unlikely that H2AX itself plays an essential role in senescence induction, since H2AX-null mice appear to have a similar lifespan to wild-type mice and maintain intact cell-cycle checkpoints [39]. MEFs taken from H2AX-null mice progress similarly toward senescence and senescent H2AX-null MEF cells exhibit characteristics similar to those from wild-type mice (data not shown). These findings indicate that the lesions marked by γ-H2AX, rather than γ-H2AX itself, are critical to the regulation of cellular senescence.

In conclusion, our results reveal that cell populations at the point of senescence appear to contain a relatively constant number of γ-foci, irrespective of their relationship to telomeres, and irrespective of whether the cells are of human or mouse origin. Thus, the total number of γ-foci at senescence appears to be independent of species or the amount of stress. These results demonstrate that the summation of different types of DNA damage may induce *in vitro *and *in vivo *aging.

## Conclusion

Both telomeric and non-telomeric related DNA damage responses are important determinants of mammalian cellular senescence. The method used in this study to quantify telomeric and non-telomeric DNA damages provides a novel approach to elucidate the processes involved in senescence and aging. The observations made in this study also have significant implications for understanding how aging changes cellular function and in designing approaches for modifying the process of cellular senescence.

## Methods

### Cell culture

WI-38 human lung fibroblasts and WI-38/SV40 (VA-13) cell lines were obtained from Coriell Cell Repositories (Camden, NJ, US) and BJ foreskin fibroblasts were obtained from ATCC (Rockville, MD, US). Human fibroblasts were grown in minimum essential medium containing 15% fetal bovine serum. TERT-null MEFs were prepared from day 13.5 embryos. The whole embryo was minced and dispersed in 0.25% trypsin and incubated for 5 minutes at 37°C. Cells were plated in T-75 flasks containing Dulbecco's minimum essential medium plus 15% fetal bovine serum. Splenocyte single cell suspensions were isolated from mTR-null mice and cultured in RPMI 1640 medium containing 20% fetal bovine serum. Splenic cells were stimulated for 48 hours using 5 μg/ml ConA, 15 μg/ml LPS and 25 CU (Cetus Unit)/ml rIL-2. Cells were maintained in a humidified incubator at 37°C, 5% CO_2 _and 20% or 3% O_2_. MEFs, grown to a pre-senescent stage under normoxic (20% O_2_) conditions were transferred to an atmosphere of 3% O_2 _and cultured to the penultimate PD, which took about 2 weeks. WI-38 cells and MEFs were incubated with 50 μM tempol for 48 hours. The 48-hour incubation time did not permit the culture to go to the penultimate stage (see Additional file 1 for experimental detail).

### PD times

WI-38 cells at 32–36 PDs, BJ cells at 35 PDs, MEFs at 3 PDs and TERT-null MEFs at 1 PD were used as low PDs. WI-38 cells at 57 PDs, BJ cells at 70–72 PDs, MEFs at 7–9 PDs and TERT-null MEFs at 9 PDs were used as high PDs [19,30,40] (see Additional file 1 for experimental detail). First generation mTR-null mice at 2 months of age and fifth generation mTR-null mice at 1 month were used as young mice. First generation mTR-null mice at 15 months and fifth generation mTR-null mice at 14 months were used as old mice.

### Mice

TERT-deficient mice and mTR-deficient mice were derived as previously described [28,29]. Deficient mice were maintained as heterozygotes on a *Mus musculus domesticus *C57BL/6(B6) background. B6 mice were originally obtained from the Frederick Cancer Research Center (Frederick, MD, US). All procedures were approved by the National Institute on Aging Animal Care and Use Committee and were in compliance with National Institutes of Health guidelines.

### Transduction of *hTERT *into WI-38 fibroblasts

Retroviral transduction of *hTERT *into WI-38 was performed as previously described [41,42]. Telomerase activity was detected in the *hTERT*-transduced WI-38, but not in control cells with the vector alone, by polymerase chain reaction-based telomere repeat amplification protocol assay. Elongated telomeres in the *hTERT*-transduced WI-38 were confirmed by Southern blot as previously described [41].

### Immunocytochemistry and FISH

Metaphase spreads were prepared as described previously [20]. Slides were stained with mouse monoclonal anti-γ-H2AX antibody (1/400 dilution, Upstate Biotechnology, Inc., Lake Placid, NY, US) followed by Alexa-488-conjugated anti-mouse immunoglobulin G (1/400 dilution, Molecular Probes, Eugene, OR, US). Then, the γ-H2AX stained cells were fixed with 50 mM ethylene glycol-bis (succinic acid N-hydroxy-succinimide ester) (Sigma, St Louis, MO, US) following telomere probe hybridization according to the telomere FISH kit (DakoCytomation, Glostrup, Denmark) protocol. DAPI (4,6-diamidino-2-phenylindole-dihydrochroride) was used for visualization of DNA. Signal was detected with an Olympus fluorescent microscope (Olympus America Inc. Melville, NY, US).

### Western blot analysis

MEFs were lysed in lysis buffer (150 mM NaCl, 20 mM hepes-NaOH (pH 7.4), 25% glycerol, 0.1 mM ethylenediaminetetraacetic acid, 0.2% NP-40, 10 mM NaF) including complete protease inhibitor (Roche, Indianapolis, IN, US). The isolated proteins were boiled in sodium dodecyl sulfate sample buffer and loaded on to 4% to 20% tris-glycine pre-cast gels (Invitrogen, Carlsbad, CA, US) The separated proteins were transferred to a polyvinylidene difluoride membrane (Invitrogen, Carlsbad, CA, US). For immunoblotting, the membranes were incubated with antibodies for p16 (1/200 dilution, Santa Cruz Biotechnology, Santa Cruz, CA, US), p21 (1/50 dilution, Abcam, Cambridge, UK), p53 (1/2000 dilution, Cell Signaling Technology, Danvers, MA, US), phospho-Ser15-p53 (1/1000 dilution, Cell Signaling Technology, Danvers, MA, US) and β-actin (1/1000 dilution, Abcam, Cambridge, UK). The blots were incubated with horseradish peroxidase-conjugated anti-mouse or anti-rabbit antibody (1/10,000 dilution, Amersham Bioscience, Piscataway, NJ, US). The blots were visualized by enhanced chemiluminescence Western blotting detection reagents (Amersham Bioscience, Piscataway, NJ, US).

## List of abbreviations

ALT: alternative lengthening of telomeres; DSB: double-strand break; FISH: fluorescence *in situ *hybridization; IM: immortalized MEF; MEF: mouse embryonic fibroblast; OX: antioxidant tempol; PD: population doubling; TERT: telomere reverse transcriptase; WS: Werner syndrome

## Authors' contributions

AJN carried out most of the analysis in this study and wrote the manuscript. YJC and KSH performed the statistical analysis and helped to draft the manuscript. IH and OAS participated in the design of the study and helped to draft the manuscript. RJH and WMB conceived and participated in the design and coordination of the study and helped to draft the manuscript. All authors read and approved the final manuscript.

## Supplementary Material

Additional file 1 Experimental design. WI-38 cells and mouse embryonic fibroblasts (MEFs) were treated with 100 ng/ml colcemid for 3 hours, and then taken for metaphase spreads. Red arrows show time points at which metaphase spreads were obtained. (A, top time line) WI-38 cells at 32 to 36 population doublings (PDs) (low PDs). WI-38 cells at 57 PDs (high PDs). (A, middle time line) WI-38 cells at 50 PDs cultured in 50 μM tempol for 48 hours (OX). (A, bottom time line) *hTERT*-transduced WI-38 cells were cultured more than 20 PDs (TERT). *hTERT *transduction was performed into WI-38 cells at 55 PDs. (B, top time line) MEFs at three PDs (low PDs). MEFs at seven PDs (high PDs 20%). (B, middle time line) MEFs cultured in 20% O_2_, shifted at seven PDs to 3% O_2 _and cultured to nine PDs (high PDs 3%). (B, bottom time line) MEFs at seven PDs cultured in 50 μM tempol for 48 hours (OX). Click here for file

Additional file 2 Amount of SA-β-gal positive cells (%) and the effect of colcemid treatment on γ-foci formation. Cells were fixed at the indicated population doublings (PDs) and SA-β-gal staining was performed following the manufacturer's instructions (Cell Signaling Technology, Danvers, MA, US). (A) Amount of SA-β-gal positive cells in WI-38 or BJ cells. (B) Amount of SA-β-gal positive cells in mouse embryonic fibroblasts (MEFs). (C) Distribution of γ-foci on metaphases of WI-38. WI-38 at 46 PDs treated with/without 100 ng/ml colcemid for 3 hours, and then taken for metaphase spreads. Proportion (%) of each type of damage is shown in each graph bar. Scoring is as in Figure 1B. On average more than 10 metaphases were screened per point in independent experiments. Error bars signify standard errors. Click here for file

Additional file 3 Experimental design of oxygen transfer experiments shown in Figure 3D. Mouse embryonic fibroblasts (MEFs) at three population doublings were cultured in 20% O_2 _or 3% O_2 _for 5 days and transferred to 3% O_2 _or 20% O_2_, respectively. Each day after transfer, MEFs were fixed and immunostained with the γ-H2AX antibody. Click here for file

Additional file 4 Distribution of senescence-related γ-foci in high population doublings of WI-38 cells and in VA-13 with alternative lengthening of telomeres. Scoring of γ-foci as along the chromatid arms proximal to the telomere, along the chromatid arms distal to the telomeres, on the chromatid ends with fluorescence *in situ *hybridization (FISH) signal or on the chromatid ends without FISH signal. Click here for file
