# Supplementary figures and images for: Both telomeric and non-telomeric DNA damage are determinants of mammalian cellular senescence
Source: Epigenetics Chromatin. 2008 Nov 3;1:6. doi: 10.1186/1756-8935-1-6 (PMC2584625; doi:10.1186/1756-8935-1-6)

**A** Human

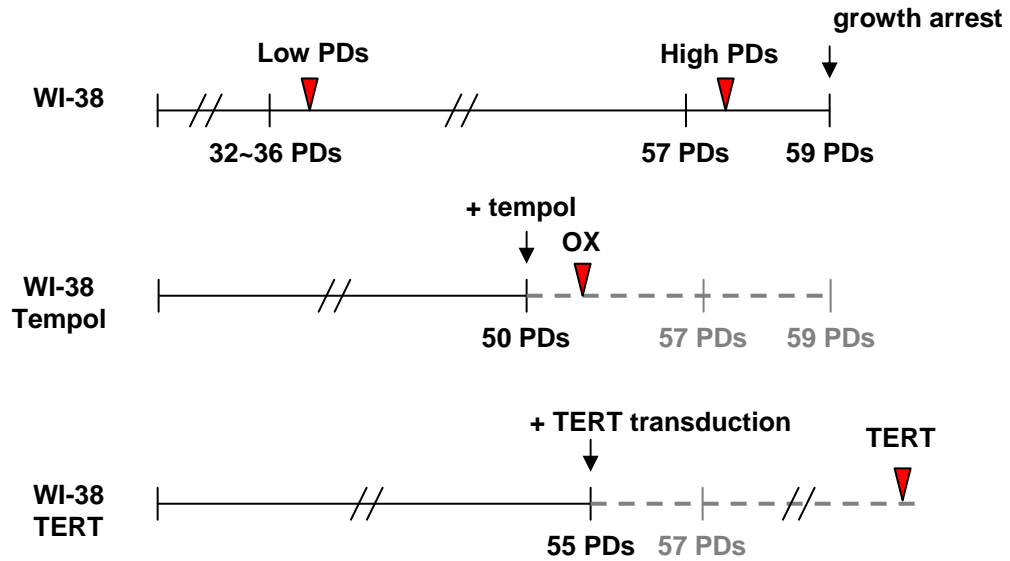

**B** Mouse

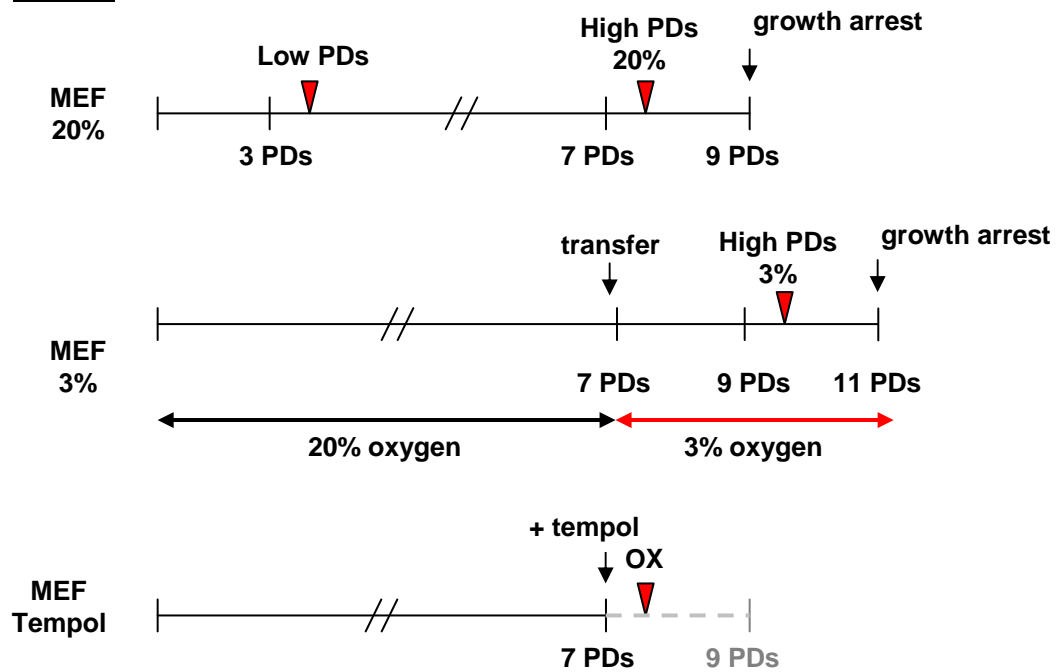

Supplement: Additional file 1 — Experimental design. WI-38 cells and mouse embryonic fibroblasts (MEFs) were treated with 100 ng/ml colcemid for 3 hours, and then taken for metaphase spreads. Red arrows show time points at which metaphase spreads were obtained. (A, top time line) WI-38 cells at 32 to 36 population doublings (PDs) (low PDs). WI-38 cells at 57 PDs (high PDs). (A, middle time line) WI-38 cells at 50 PDs cultured in 50 μM tempol for 48 hours (OX). (A, bottom time line) hTERT-transduced WI-38 cells were cultured more than 20 PDs (TERT). hTERT transduction was performed into WI-38 cells at 55 PDs. (B, top time line) MEFs at three PDs (low PDs). MEFs at seven PDs (high PDs 20%). (B, middle time line) MEFs cultured in 20% O2, shifted at seven PDs to 3% O2 and cultured to nine PDs (high PDs 3%). (B, bottom time line) MEFs at seven PDs cultured in 50 μM tempol for 48 hours (OX). [file 1756-8935-1-6-S1.pdf]

**A**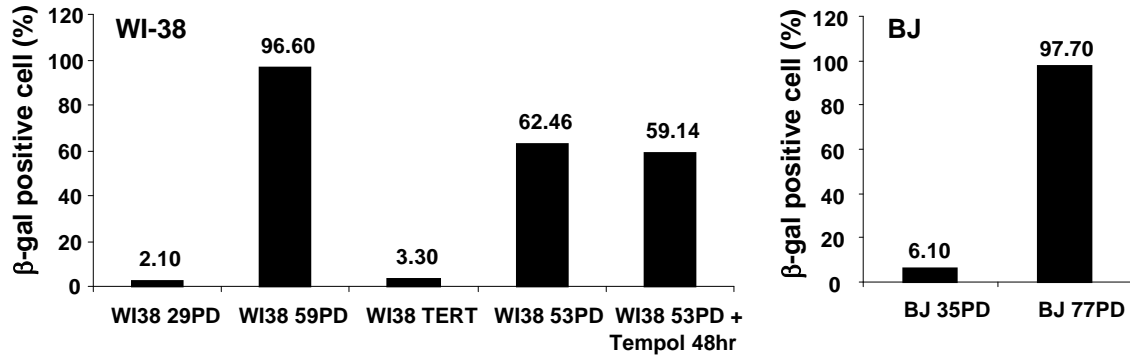**B**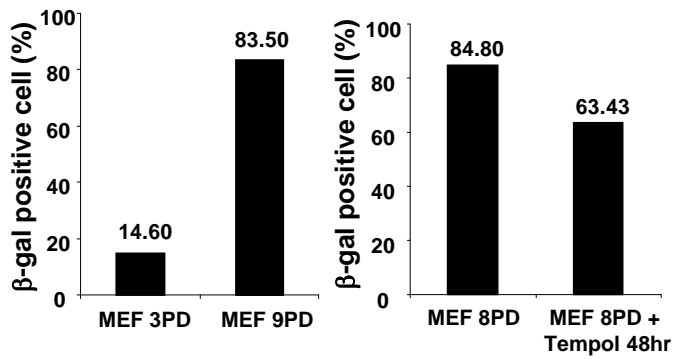**C**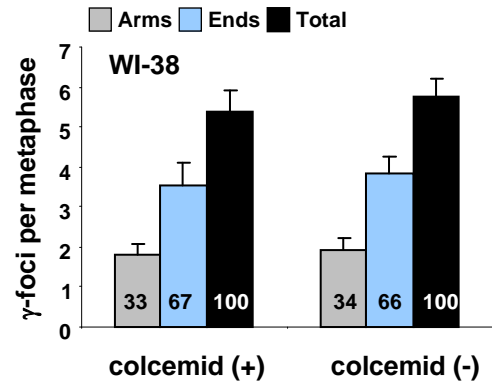

Supplement: Additional file 2 — Amount of SA-β-gal positive cells (%) and the effect of colcemid treatment on γ-foci formation. Cells were fixed at the indicated population doublings (PDs) and SA-β-gal staining was performed following the manufacturer's instructions (Cell Signaling Technology, Danvers, MA, US). (A) Amount of SA-β-gal positive cells in WI-38 or BJ cells. (B) Amount of SA-β-gal positive cells in mouse embryonic fibroblasts (MEFs). (C) Distribution of γ-foci on metaphases of WI-38. WI-38 at 46 PDs treated with/without 100 ng/ml colcemid for 3 hours, and then taken for metaphase spreads. Proportion (%) of each type of damage is shown in each graph bar. Scoring is as in Figure 1B. On average more than 10 metaphases were screened per point in independent experiments. Error bars signify standard errors. [file 1756-8935-1-6-S2.pdf]

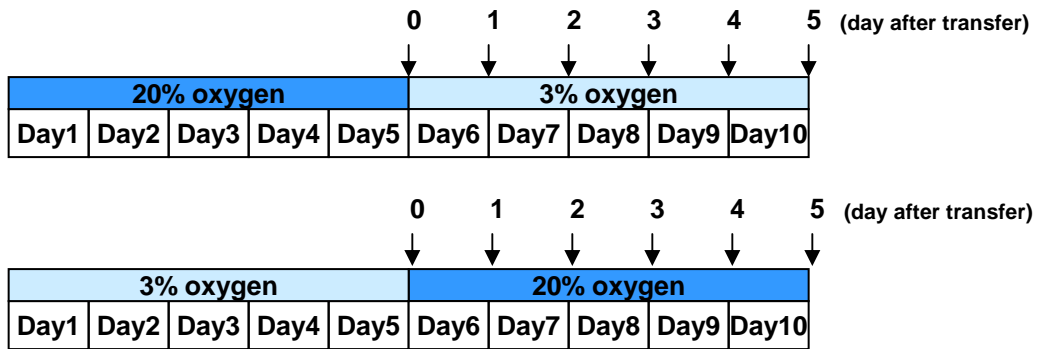

Supplement: Additional file 3 — Experimental design of oxygen transfer experiments shown in Figure 3D. Mouse embryonic fibroblasts (MEFs) at three population doublings were cultured in 20% O2 or 3% O2 for 5 days and transferred to 3% O2 or 20% O2, respectively. Each day after transfer, MEFs were fixed and immunostained with the γ-H2AX antibody. [file 1756-8935-1-6-S3.pdf]

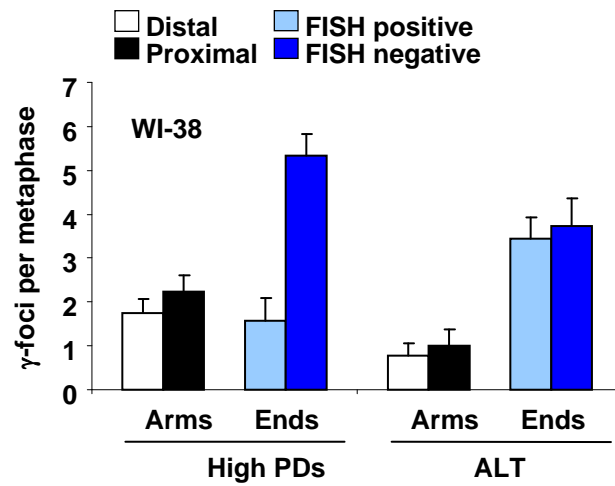

Supplement: Additional file 4 — Distribution of senescence-related γ-foci in high population doublings of WI-38 cells and in VA-13 with alternative lengthening of telomeres. Scoring of γ-foci as along the chromatid arms proximal to the telomere, along the chromatid arms distal to the telomeres, on the chromatid ends with fluorescence in situ hybridization (FISH) signal or on the chromatid ends without FISH signal. [file 1756-8935-1-6-S4.pdf]
